# Supplementary figures and images for: Is coral richness related to community resistance to and recovery from disturbance?
Source: PeerJ. 2014 Mar 18;2:e308. doi: 10.7717/peerj.308 (PMC3970800; doi:10.7717/peerj.308)

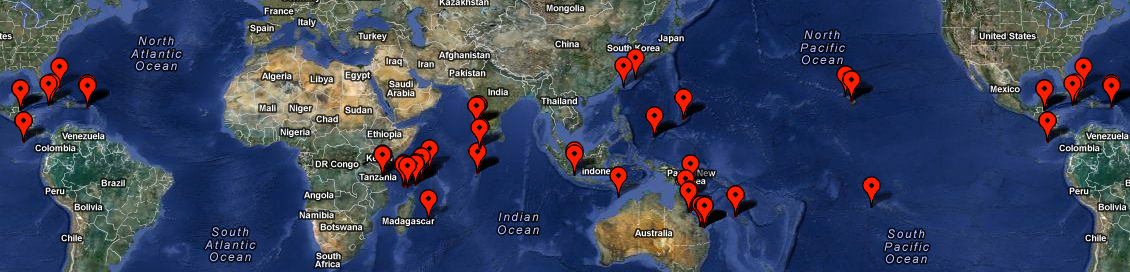

Supplement: Figure S1 — Also see the live version in Google Maps. [file peerj-02-308-s001.png]

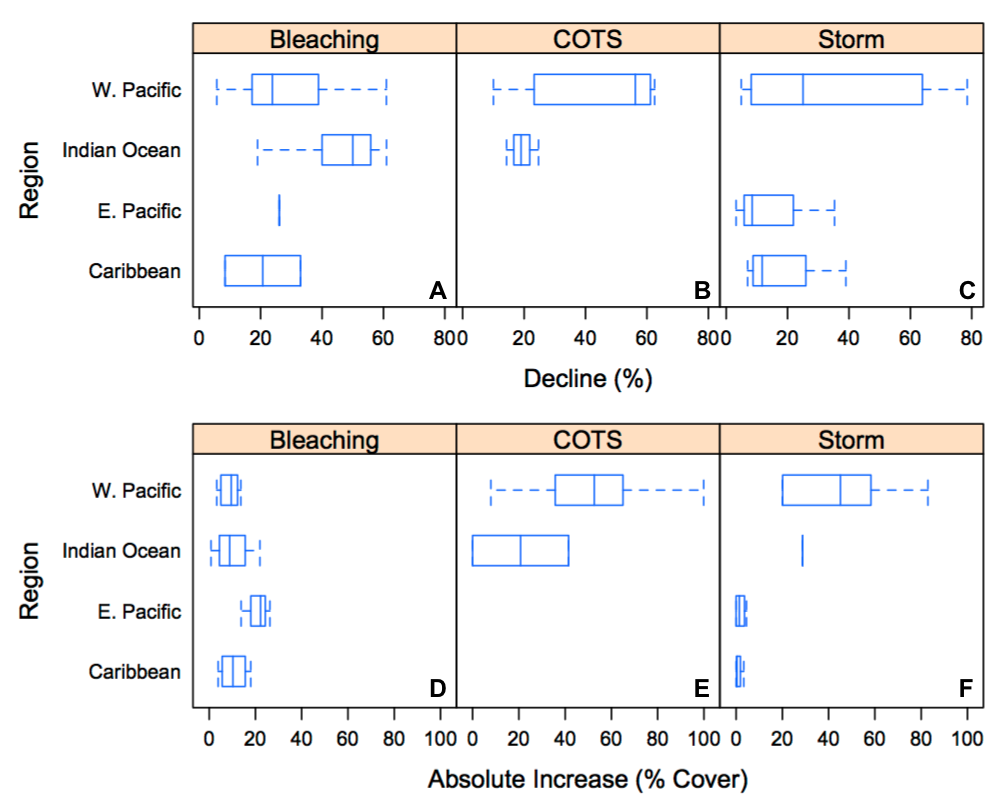

Supplement: Figure S2 — Comparisons of coral cover decline from the resistance analysis (A–C) and post-disturbance coral cover increase from the recovery analysis (D–F) among regions and disturbance types. Boxes are interquartile ranges and lines within each box represent median values. Error bars extend to the minimum and maximum values. [file peerj-02-308-s002.png]

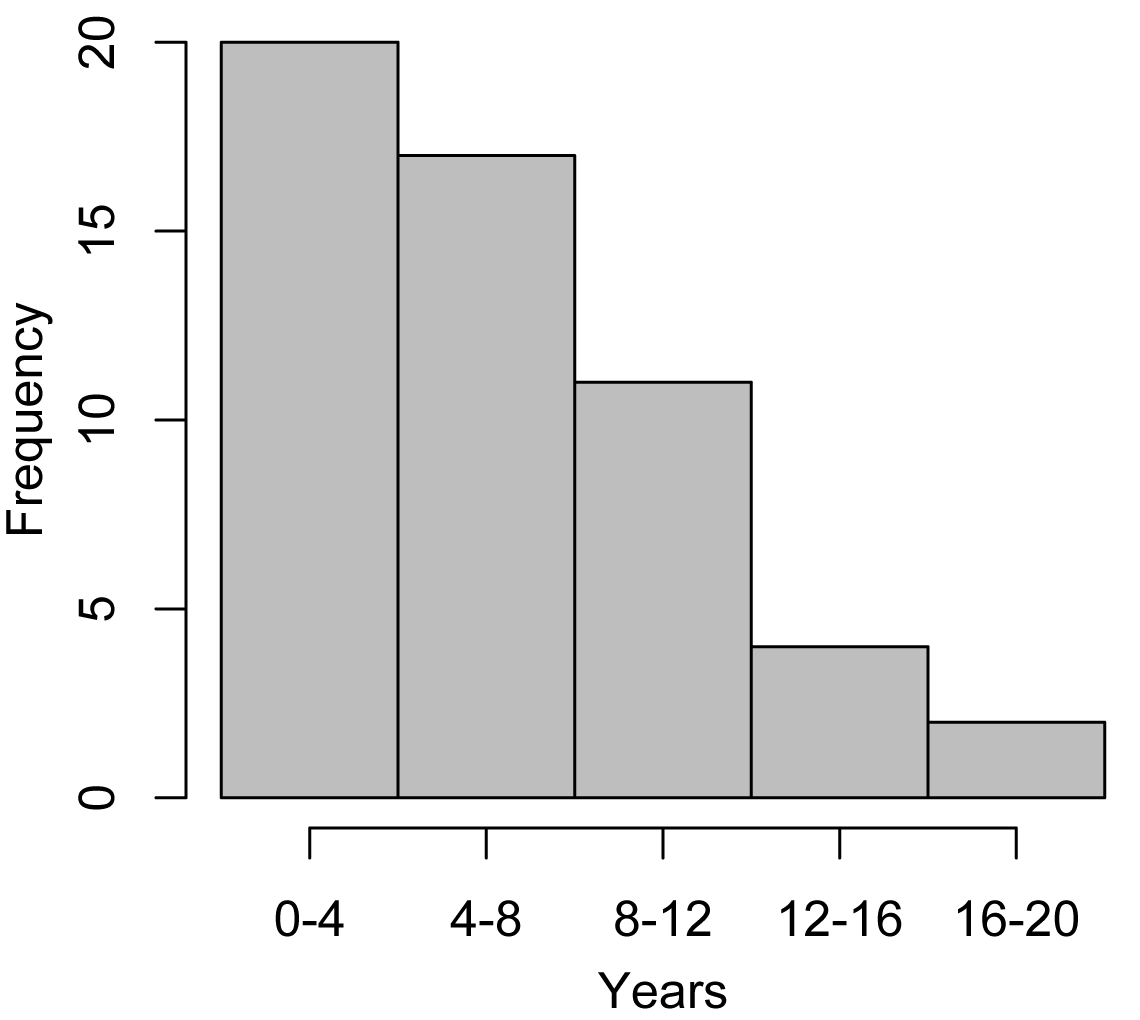

Supplement: Figure S3 [file peerj-02-308-s003.png]
