# Supplementary material for: Is coral richness related to community resistance to and recovery from disturbance?
Source: PeerJ. 2014 Mar 18;2:e308. doi: 10.7717/peerj.308 (PMC3970800; doi:10.7717/peerj.308)
Supplement: Supplemental Information 4 [file peerj-02-308-s004.rtf]

This metadata is for the file "reefresilience_data.csv" which is the core dataset for Zhang et al. See manuscript for details regarding the study purpose and data collection. Briefly, we tested the hypothesis that coral species richness and other covariates are related to the loss of coral cover due to disturbances and subsequent coral community recovery. Data columns:study=data source (refer to Text S1 for full citations)disturbance=categorical disturbance typeregion=categorical geographical regionmindepth, maxdeopth=min and max depth (m) as reported in the published studies blcc="baseline" or pre-disturbance total absolute living coral coverpdcc=post-disturbance living coral coverdecline=coral cover change recorded and attributed to observed disturbance by component studies, i.e., blcc - pdcctot.rec=total coral cover recovery; observed change (generally an increase) in coral cover after disturbance by the end of the studyyears.bw=number of years until peak post-disturbance coral cover was recordedrichness=estimated coral species richness rec.rate=rate of annual increase in coral cover change post-disturbance (tot.rec/years.bw)
