# Supplementary material for: Is coral richness related to community resistance to and recovery from disturbance?
Source: PeerJ. 2014 Mar 18;2:e308. doi: 10.7717/peerj.308 (PMC3970800; doi:10.7717/peerj.308)
Supplement: Statistical Supplement — Complete R code, model outputs, etc. for the primary analyses [file peerj-02-308-s006.docx]

**Statistical supplement for Zhang et al.**

Data file: “reefresilience_data_final.csv”

1) check variance inflation factors (VIFS of continuous predictors):


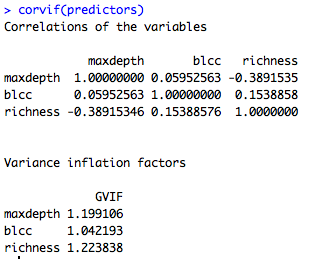


Richness and blcc are not related:


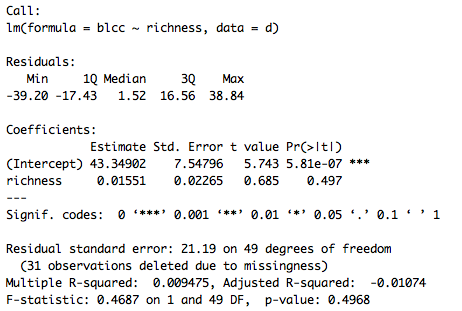


And blcc does not vary among regions:


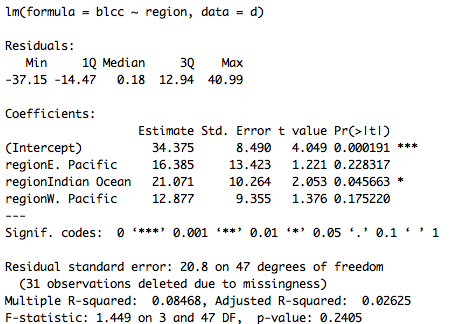


2) Explore decline data:


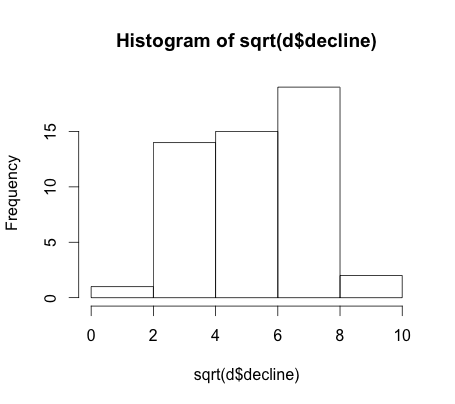

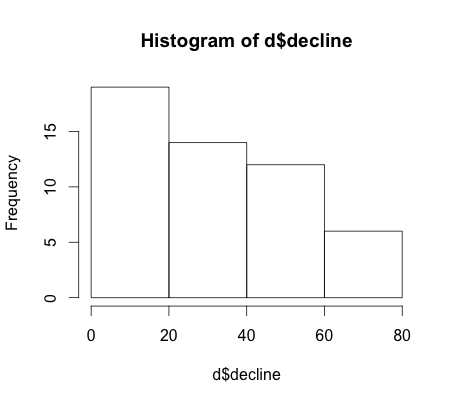


Sqrt transformed data appears more normal.

3) Is richness related to post-disturbance coral cover loss (sqrt-transformed decline)?

Model 1: full decline model with all five covariates


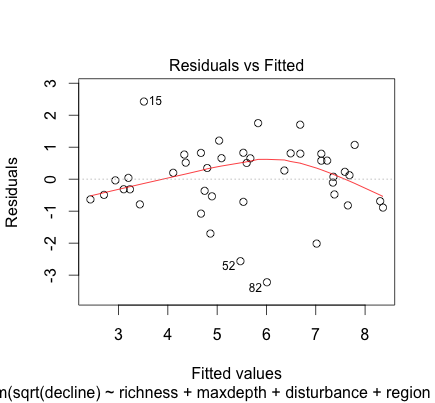

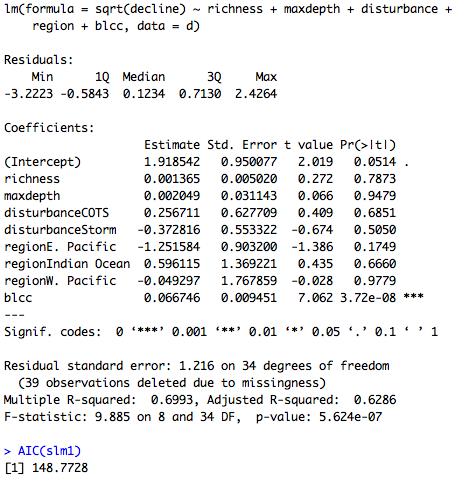


Model 2: reduced model without disturbance and region


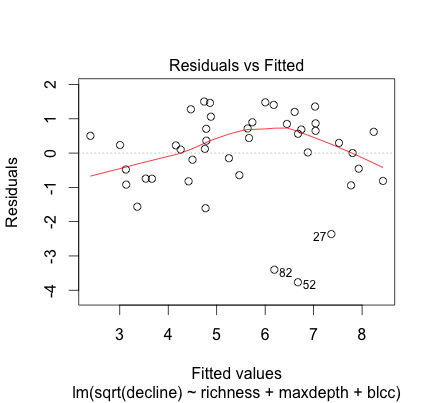

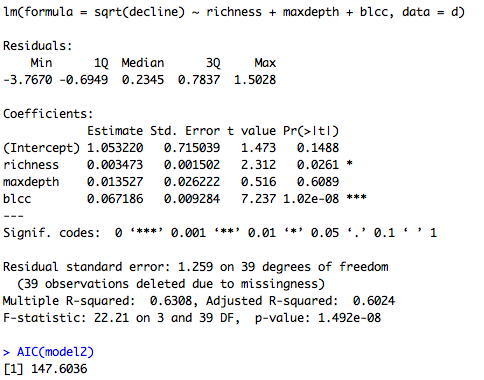


Compare Model 1 and 2 (fit does not differ based on comparison of AIC)


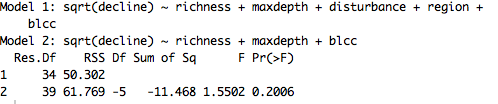


Model 3: only richness and blcc included as covariates


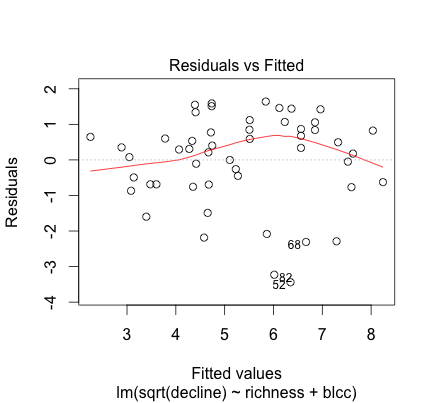

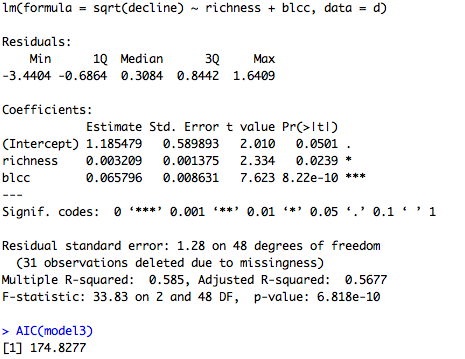


Adding interaction terms did not improve model fit (and terms were not significant, e.g., depth x disturbance), e.g.;


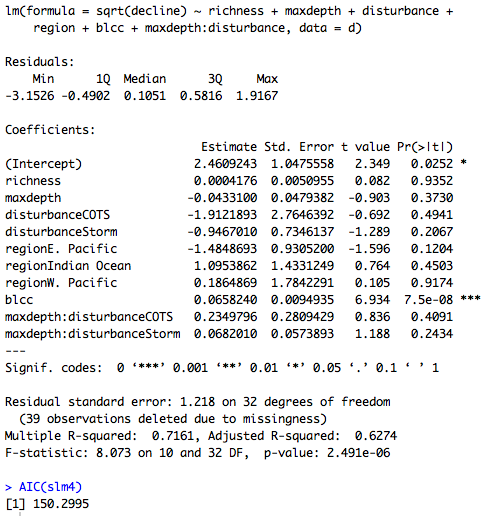


4) Explore recovery data:


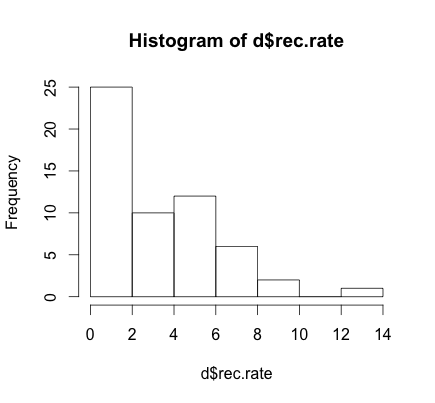

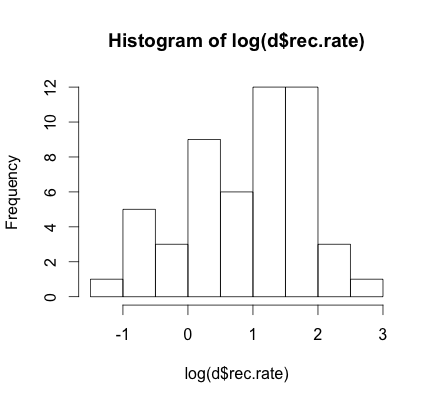


Raw data are very skewed with a fair number of very low values. Log transformed data appears more normal.

5) Is richness related to recovery?

Model 4: full recovery model on log transformed recovery rate with all five covariates


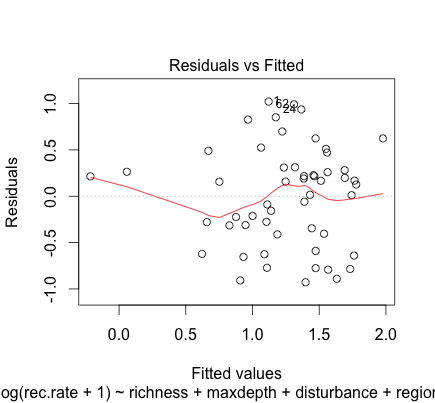

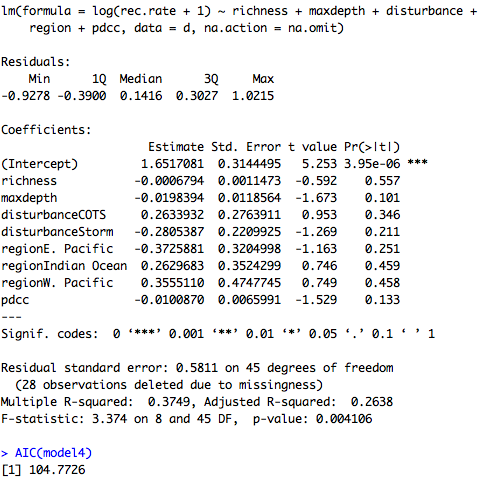


Model 5: without disturbance and region


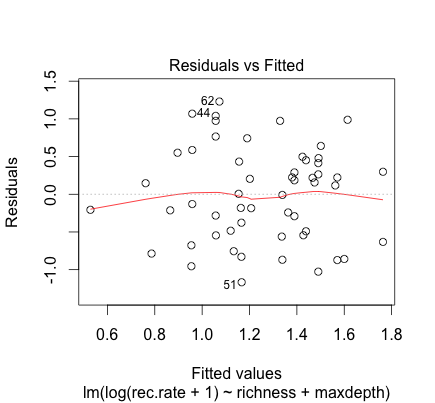

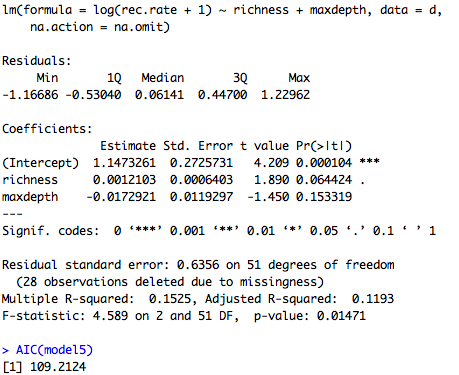


AIC of model 4 is significantly lower than that of model 5:


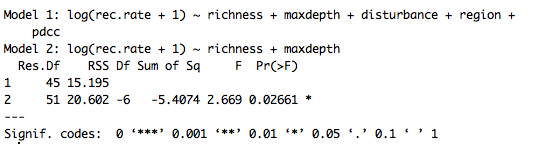


Model 6: without depth, disturbance, and region (only richness as a covariate)


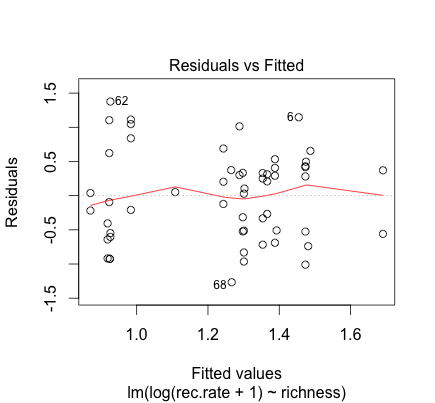


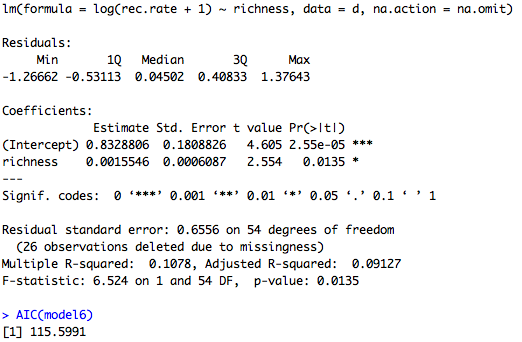


Richness effect becomes significant but AIC goes up.

Below are versions of the two main figures in the manuscript with transformed response variables plotted instead of raw data.


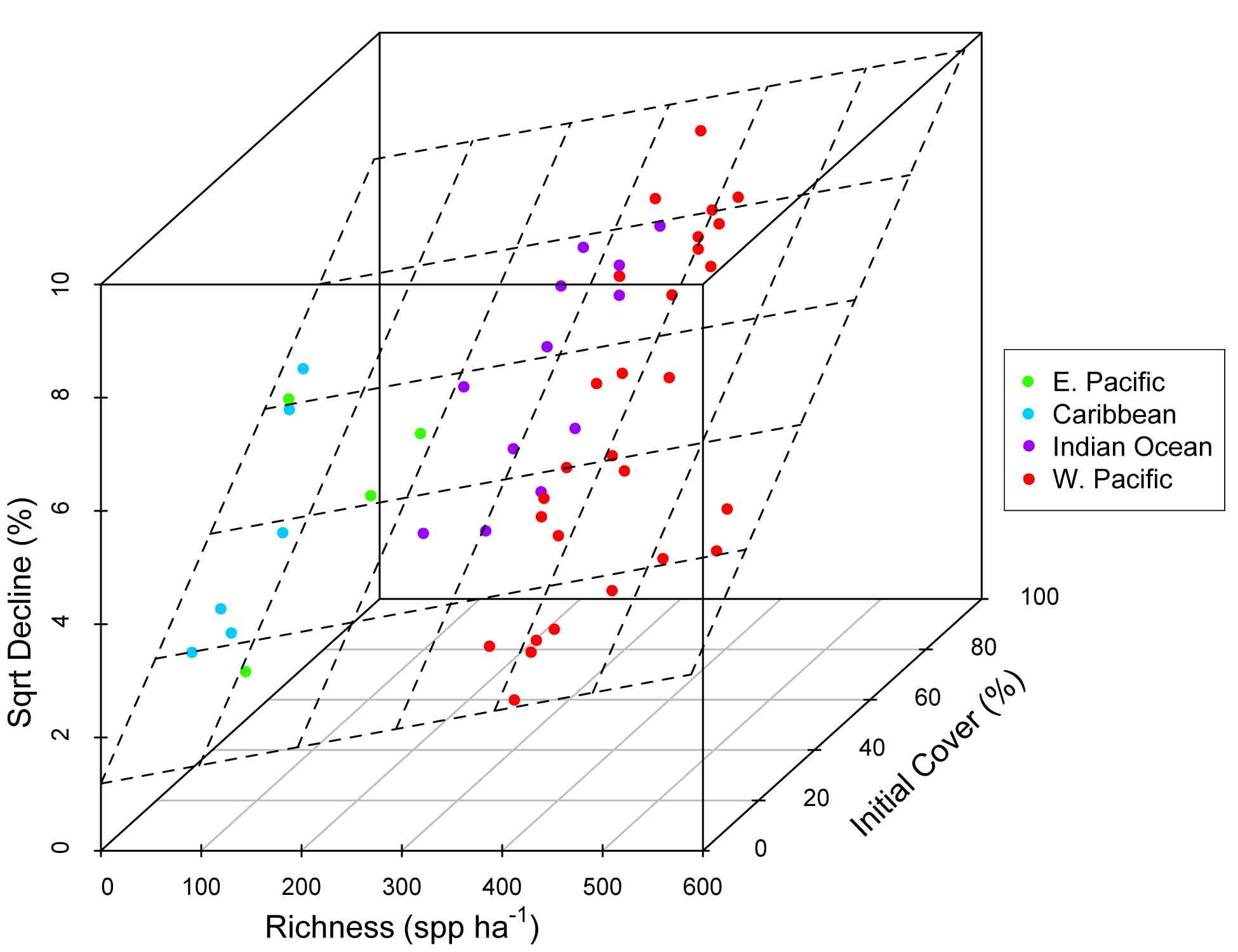


3-D scatter plot of the relationship between coral community richness, coral loss (Sqrt net change in absolute percent cover in response to disturbance) and initial coral cover. Dashed lines represent the regression plane, and point coloration represents region.


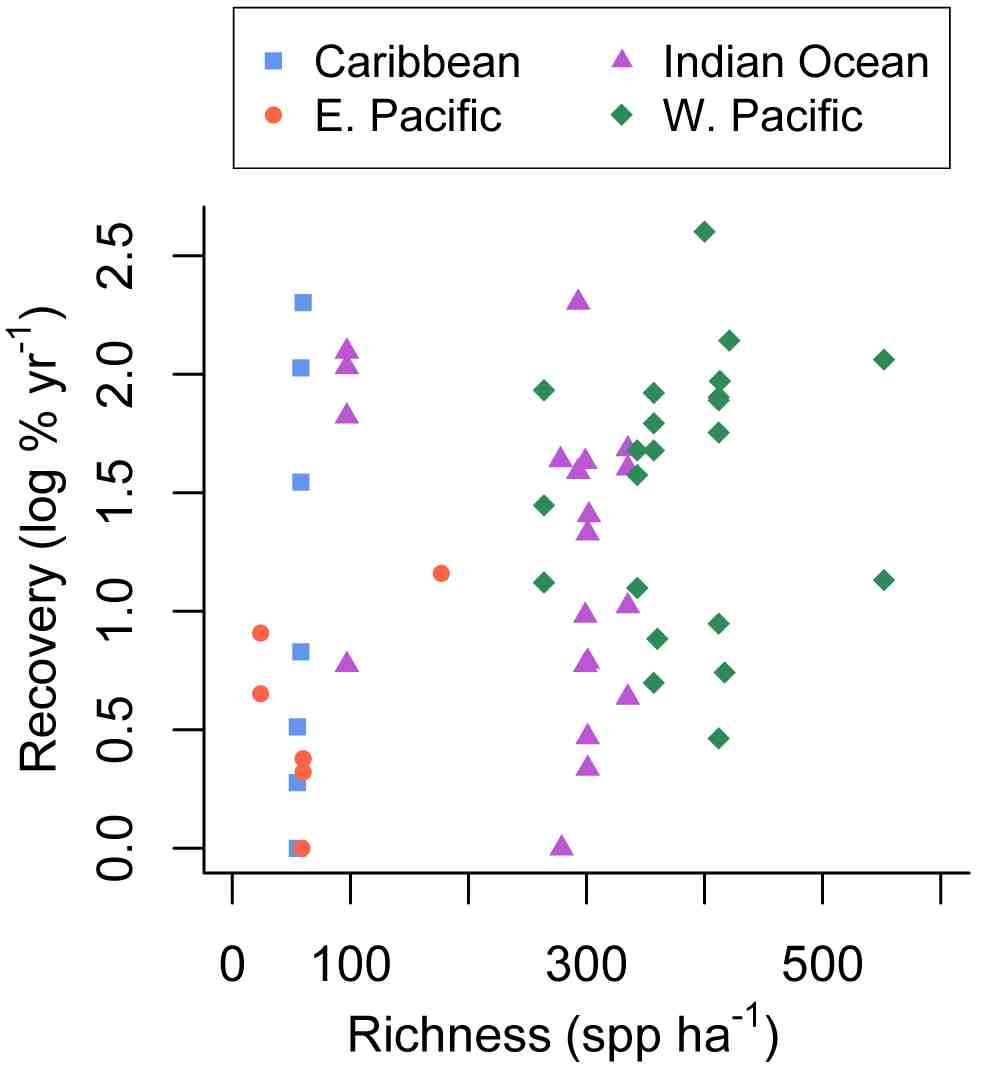


Scatter plot of the relationship between log post-disturbance recovery of coral cover and estimated coral species richness.
