# Supplementary material for: Is coral richness related to community resistance to and recovery from disturbance?
Source: PeerJ. 2014 Mar 18;2:e308. doi: 10.7717/peerj.308 (PMC3970800; doi:10.7717/peerj.308)
Supplement: Text S1 [file peerj-02-308-s007.docx]

Text S1. Data sources.

Adjeroud, M., et al. (2009). "Recurrent disturbances, recovery trajectories, and resilience of coral assemblages on a South Central Pacific reef." Coral Reefs 28(3): 775-780.

Arthur, R., et al. (2006). "Local processes strongly influence post-bleaching benthic recovery in the Lakshadweep Islands." Coral Reefs 25(3): 427-440.

Booth, D. J. and G. A. Beretta (2002). "Changes in a fish assemblage after a coral bleaching event." Marine Ecology Progress Series 245: 205-212.

Brown, B. E. and S. Suharsono (1990). "Damage and recovery of coral reefs affected by El Niño related seawater warming in the Thousand Islands, Indonesia." Coral Reefs 8(4): 163-170.

Buckley, R., et al. (2008). Was the 1998 coral bleaching in the Southern Seychelles a catastrophic disturbance? - 1999-2006 Reef Fish Responses to Coral Substrate Changes. Proceedings of the 11th International Coral Reef Symposium (ICRS 2008).

Coles, S. and E. Brown (2007). "Twenty-five years of change in coral coverage on a hurricane impacted reef in Hawai‘i: the importance of recruitment." Coral Reefs 26(3): 705-717.

Colgan, M. W. (1987). "Coral reef recovery on Guam (Micronesia) after catastrophic predation by Acanthaster planci." Ecology 68: 1592-1605.

Connell, J. H., et al. (1997). "A 30-year study of coral abundance, recruitment, and disturbance at several scales in space and time." Ecological Monographs 67(4): 461-488.

Crabbe, M. J. C. (2009). "Scleractinian coral population size structures and growth rates indicate coral resilience on the fringing reefs of North Jamaica." Marine Environmental Research 67(4–5): 189-198.

Dollar, S. J. (1982). "Wave stress and coral community structure in Hawaii." Coral Reefs 1: 71-81.

Dollar, S. J. and G. W. Tribble (1993). "Recurrent storm disturbance and recovery: a long-term study of coral communities in Hawaii." Coral Reefs 12(3): 223-233.

Done, T., et al. (2007). "Decadal changes in turbid-water coral communities at Pandora Reef: loss of resilience or too soon to tell?" Coral Reefs 26(4): 789-805.

Done, T. J. (1985). “Effects of two Acanthaster outbreaks on coral community structure - the meaning of devastation.” The Fifth International Coral Reef Congress, Tahiti.

Done, T. J., et al. (1988). Recovery of coral communities post Acanthaster: Progress and prospects. Proceedings of the 6th International Coral Reef Symposium (ICRS 1988). 2: 137-142

Edmunds, P. J. (2002). "Long-term dynamics of coral reefs in St. John, US Virgin Islands." Coral Reefs 21(4): 357-367.

Edwards, A. J., et al. (2001). "Coral bleaching and mortality on artificial and natural reefs in Maldives in 1998, sea surface temperature anomalies and initial recovery." Marine Pollution Bulletin 42(1): 7-15.

Emslie, M., et al. (2008). "Recovery from disturbance of coral and reef fish communities on the Great Barrier Reef, Australia." Marine Ecology Progress Series 371: 177-190.

Golbuu, Y., et al. (2007). "Palau’s coral reefs show differential habitat recovery following the 1998-bleaching event." Coral Reefs 26(2): 319-332.

Guillemot, N., et al. (2010). "Cyclone effects on coral reef habitats in New Caledonia (South Pacific)." Coral Reefs 29(2): 445-453.

Guillermo Diaz-Pulido, et al. (2009). "Doom and boom on a resilient reef: climate change, algal overgrowth and coral Recovery." PLoS ONE 4(4): e5239.

Guzman HM, Cortés J (2007) “Reef recovery 20 years after the 1982–1983 El Nino massive mortality.” Marine Biology 151:401–411

Hagan, A. and T. Spencer (2008). “Reef Resilience and Change 1998-2007, Alphonse Atoll, Seychelles.” 11th International Coral Reef Symposium (ICRS 2008).

Halford, A., et al. (2004). "Resilience to large-scale disturbance in coral and fish assemblages on the Great Barrier Reef." Ecology 85(7): 1892-1905.

Loya, Y., et al. (2001). "Coral bleaching: the winners and the losers." Ecology Letters 4(2): 122-131.

McClanahan, T. R. (2000). "Bleaching damage and recovery potential of Maldivian coral reefs." Marine Pollution Bulletin 40(7): 587-597.

Muhando, C. A. L., F. (2008). “Ecological effects of the crown-of-thorns starfish removal programme on Chumbe Island Coral Park, Zanzibar, Tanzania.” Proceedings of the 11th International Coral Reef Symposium (ICRS 2008).

Naim, O. C., P.; Done, T.; Tourrand, C. and Letourner, Y. (2000). “Regeneration of a reef flat ten years after the impact of the cyclone Firinga (Reunion, SW Indian Ocean).” Proceedings of the 9th International Coral Reef Symposium (ICRS 2000).

Ostrander, G. K., et al. (2000). "Rapid transition in the structure of a coral reef community: The effects of coral bleaching and physical disturbance." Proceedings of the National Academy of Sciences 97(10): 5297-5302.

Pratchett, M. S. (2010). "Changes in coral assemblages during an outbreak of Acanthaster planci at Lizard Island, northern Great Barrier Reef (1995–1999)." Coral Reefs 29(3): 717-725.

Pratchett, M. S., et al. (2008). Effects of climate-induced coral bleaching on coral-reef fishes — ecological and economic consequences. Oceanography and marine biology: an annual review. R. N. Gibson, R. J. A. Atkinson and J. D. M. Gordon, CRC Press. 46: 251-296.

Pratchett, M. S., et al. (2009). "Selective coral mortality associated with outbreaks of Acanthaster planci L. in Bootless Bay, Papua New Guinea." Marine Environmental Research 67(4–5): 230-236.

Robbart, M. L., et al. (2004). "Population recovery and differential heat shock protein expression for the corals Agaricia agaricites and A. tenuifolia in Belize." Marine Ecology Progress Series 283: 151-160.

Rogers, C. S. and J. Miller (2006). "Permanent 'phase shifts' or reversible declines in coral cover? Lack of recovery of two coral reefs in St. John, US Virgin Islands." Marine Ecology Progress Series 306: 103-114.

Rogers, C. S., et al. (1982). "Effects of Hurricanes David and Frederic (1979) on shallow Acropora palmata reef communities: St. Croix, U. S. Virgin Islands." Bulletin of Marine Science 32: 532-548.

Sano, M. (2000). "Stability of reef fish assemblages: responses to coral recovery after catastrophic predation by Acanthaster planci." Marine Ecology Progress Series 198: 121-130.

Sheppard, C. R. C., et al. (2008). "Archipelago-wide coral recovery patterns since 1998 in the Chagos Archipelago, central Indian Ocean." Marine Ecology Progress Series 362: 109-117.

Smith, L., et al. (2008). "Resilience of coral communities on an isolated system of reefs following catastrophic mass-bleaching." Coral Reefs 27(1): 197-205.

Spalding, M. D. and G. E. Jarvis (2002). "The impact of the 1998 coral mortality on reef fish communities in the Seychelles." Marine Pollution Bulleting 44(4): 309-321.

Stobart, B., et al. (2002). Aldabra Marine Program. Phase III. Extending the survey eastward., Cambridge Coastal Research Unit, University of Cambridge.

Stobart, B., et al. (2005). "Coral recovery at Aldabra Atoll, Seychelles: Five years after the 1998 bleaching event." Philosophical Transactions: Mathematical, Physical and Engineering Sciences 363(1826): 251-255.

Woodley, J. D., et al. (1981). "Hurricane Allen's impact on Jamaican coral reefs." Science 214: 749-755.
